# Automated monitoring the kinetics of homogeneous and heterogeneous chemical processes using a smartphone

**DOI:** 10.1038/s41598-022-20123-9

**Published:** 2022-09-21

**Authors:** Mateus H. Keller, Raphaell Moreira, Bruno S. Souza

**Affiliations:** 1 Department of Chemistry, Federal University of Santa Catarina, Florianopolis, Brazil; 2 Department of Chemical and Biological Engineering, The University of British Columbia, 2360 East Mall, Vancouver, BC V6T 1Z3 Canada

**Keywords:** Catalysis, Chemical education, Inorganic chemistry, Organic chemistry, Physical chemistry

## Abstract

Heterogeneous chemical processes occupy a pivotal position in many fields of applied chemistry. Monitoring reaction kinetics in such heterogeneous systems together with challenges associated with ex-situ analytical methodologies can lead to inaccurate information about the nature of the catalyst surfaces as well as information about the steps involved. The present work explores the possibility of kinetic measurements of chemical reactions and adsorption processes of homogeneous and heterogeneous systems through the variation of RGB intensities of digital images using a smartphone combined with a program written in Python to accelerate and facilitate data acquisition. In order to validate the method proposed, the base promoted hydrolysis of 4-nitrophenyl acetate was initially investigated. The rate constants obtained through RGB analysis (0.01854 min^−1^) is almost identical to that using traditional UV–Vis spectroscopy (0.01848 min^−1^). The proposed method was then applied to monitor the kinetics of three heterogeneous processes: (1) reduction of 4-nitrophenolate in the presence of dispersed Pd/C; (2) decomposition of methyl orange with TiO_2_; and (3) adsorption of rhodamine on montmorillonite. In general, the method via digital images showed high reproducibility and analytical frequency, allowing the execution of simultaneous analyses, with an accuracy comparable to UV–Vis spectrophotometry. The method developed herein is a practical and valuable alternative for obtaining kinetic data of heterogeneous reactions and processes where a color change is involved, bypassing sampling collection and processing which decreases analytical frequency and may lead to data errors.

## Introduction

Chemical kinetics is the branch of chemistry responsible for studying the rate of chemical processes^1^. These studies provide useful information which can, for example, be used to suggest a reaction mechanism. Moreover, knowing the reaction kinetic law of a given system allows one to rationally change experimental variables, such as temperature, concentration or pressure, aiming to maximize the speed of product formation. Besides, kinetic measurements are at the heart of catalyst development and testing. Therefore, measuring the rate of chemical reactions is of paramount importance both in academia and industry.

Several experimental techniques can be employed to monitor reaction kinetics. Electroanalytical, chromatographic, spectroscopic, and even the most classical techniques, such as acid–base titration and pressure variation measurements, are employed. Essentially, any method that allows to monitor the concentration of reagents or products as a function of time can be used to determine the rate of a reaction^2^.

The most common spectroscopic technique employed in routine kinetics studies is Ultraviolet–Visible Molecular Absorption Spectrophotometry (UV–Vis). This method is popular because of the use of low reagent concentrations, operational simplicity and the ability to automate kinetic measurements using commercially available equipment^3,4^.

In UV–Vis kinetic monitoring the absorbance at a given wavelength is monitored as a function of time. Although it works perfectly in homogeneous media, the UV–Vis technique presents problems for heterogeneous solutions by the simple fact that particles in solution deviate the incident radiation, so that the transmitted radiation intensity is inconsistent with the actual absorption of the sample. Thus, if the UV–Vis kinetic monitoring of a heterogeneous system is wanted, a particulate removal step, such as filtration or centrifugation, prior to spectrum acquisition, is often required. This sample processing step increases analysis time, hampers automation, and can lead to measurement errors. Faced with this fact, there is a frequent search for more routine, fast and real-time monitoring methods.

Recent advances in technology have enabled the development of automated platforms that aid in decreasing hands-on experimental time and increase throughput, facilitating the screening of reaction variables. While effective when applied to kinetic studies for the development of new catalysts and for the understanding of chemical reactions^5–7^, for example, most existing systems are only partially automated and still require human intervention and only a few studies on kinetic monitoring by digital images were reported thus far^8–10^.

The use of smartphones for analytical purposes has been widely exploited by researchers in the past decade. Smartphones can be used for the development of portable electrochemical biosensors using circuit boards with Bluetooth technology^11,12^. In addition, it possible to exploit the smartphone built-in camera as a detection tool. Traditional digital cameras collect data in red, green, and blue (RGB) color channels, corresponding to pixels that are sensitive to light at wavelengths of approximately 620, 550, and 450 nm, respectively. Therefore, the ubiquity of cell phone cameras makes them an accessible tool to monitor color changes as a proxy to absorbance as a function of time. This has been explored in many different application, such as for analysis of wine^13,14^, ketchup^15^, detection of explosives^16,17^, detection of bacteria^18^, etc.^19–21^. A great number of studies on the currently applications of smartphones in analytical chemistry is reviewed elsewhere^22,23^.

An object that absorbs radiation in the visible range presents color, which is perceived by the detector (e.g. human eyes or camera) as a result of the light that is transmitted or scattered from it. Specifically speaking in the case of a colored solution, a certain part of the incident light spectrum is absorbed, whereas the human eye perceives the color from the solution as the complementary color of the radiation absorbed. In heterogeneous systems composed of small and sparse particles suspended the color is a result of the transmitted light from the medium, and in the case of opaque systems, where the high concentration of particles does not permit that visible light passes through the medium, the observed color is a result of the scattering of the radiation that is not absorbed by the medium. Thus, the color of an object is directly related to its chemical composition.

Considering the problems associated with kinetics analysis of heterogeneous systems by UV–Vis spectroscopy and the possibility of developing an alternative methodology for kinetic reaction monitoring, the motivation of this work is to develop a simple, automated, fast and frugal innovation for monitoring solution kinetics using a smartphone. To the best of our knowledge, real time monitoring of chemical reactions from images obtained with an ordinary smartphone has not been explored yet.

## Results and discussion

### Process overview

**Figure 1:**
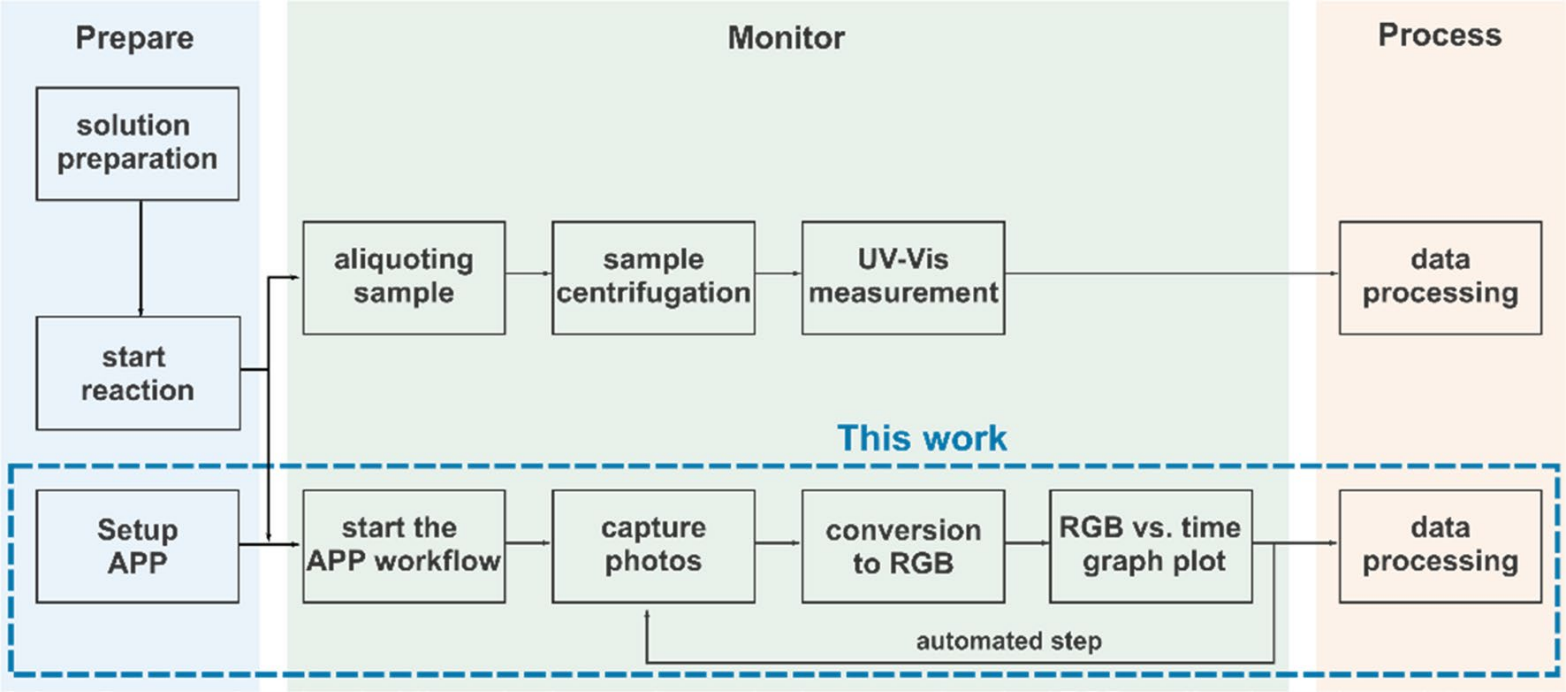
Experimental kinetic monitoring workflow overview.

The methodology developed herein to monitor chemical kinetics using a smartphone is based on the workflow given in Fig. 1. Before starting the chemical process, the application developed in this work (details in Supplementary Information S1) is programed with the desired parameters, such as time interval between data acquisition and image selected area. Once the kinetic is triggered, which is can be done by the addition of a reactant, the system automatically records and plots RGB values as a function of time. For the sake of comparison, aliquots can be taken for traditional analysis using UV–Vis. If particulates are present, a filtration or centrifugation step is needed prior to UV–Vis analysis.

### Validation of experimental setup: hydrolysis of 4-NPA

**Figure 2:**
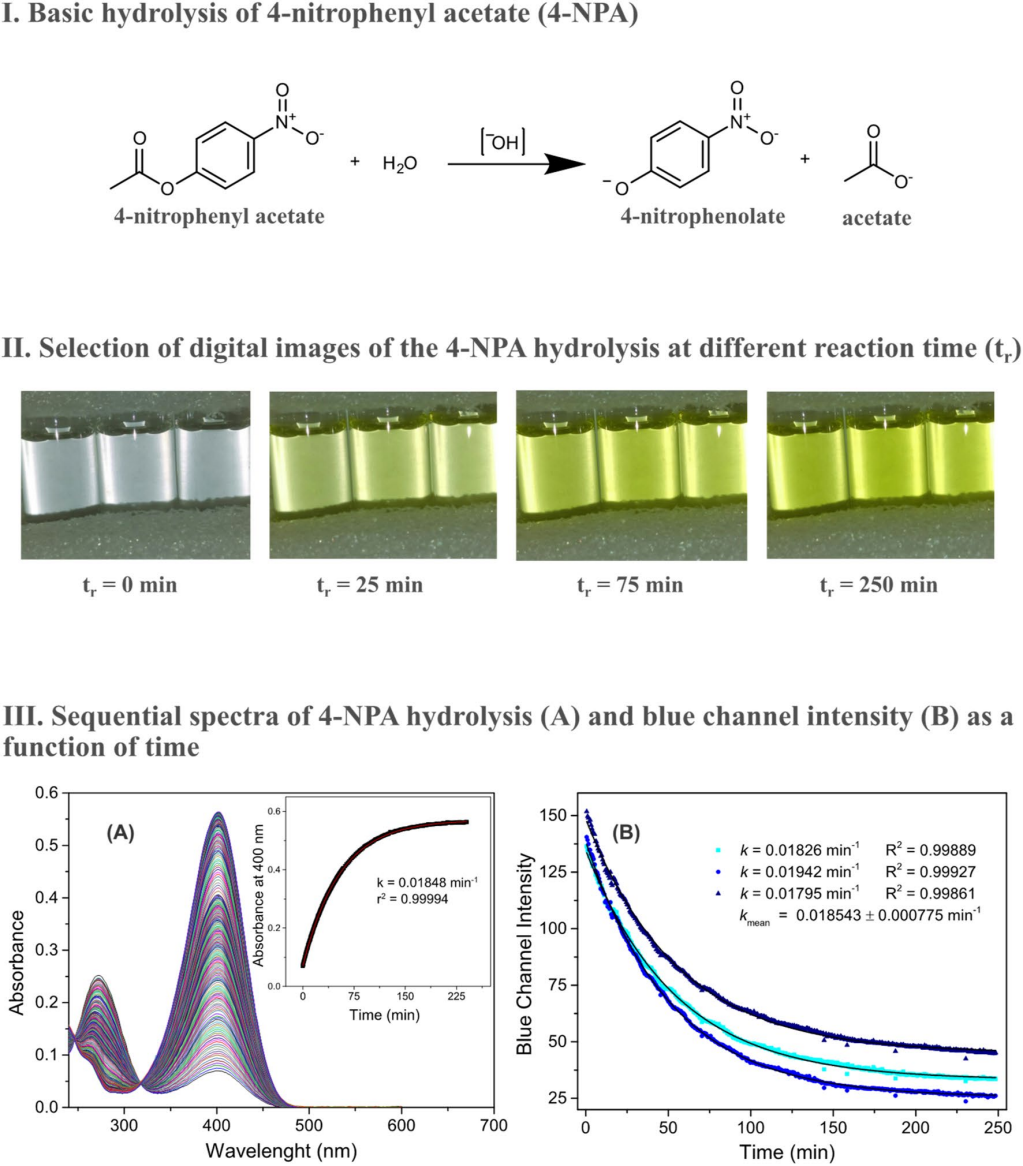
Base promoted hydrolysis of 4-NPA (I). Selection of digital images used in 4-NPA hydrolysis monitoring (II). Successive scan spectra for the hydrolysis of 4-nitrophenyl acetate and absorbance profile at 400 nm as a function of time (III-**A**); blue intensity profile as a function of reaction time (III-**B**).

To validate our experimental setup, we initially studied the homogenous base promoted hydrolysis of the 4-NPA substrate to 4-nitrophenolate (4-NP) (Fig. 2). This substrate is widely used to study the catalytic activity of esterases^24^ and new artificial hydrolytic systems^25^ because the leaving group 4-NP absorbs strongly at 400 nm.

Prior to the kinetic monitoring, we confirmed that the 4-NP concentration is linearly correlated with the Blue channel, while the Red and Green channels intensities remain relatively constant (Fig. S1). In this experiment, a colorless medium (aqueous basic solution) placed in front of a white background turns yellowish by the addition or formation, in the case of 4-NPA hydrolysis, of 4-NP. Ideally, before 4-NP is present the photo acquired from the solution would be pure white, i.e., when no dye is present it is expected that RGB values are all 255. As 4-NP is added the color detected should become yellowish. For pure yellow R = 255, G = 255 and B = 0. However, the actual color acquired in the initial system is grayish, with lower RGB values than white, and the yellowish final solution has lower RG and higher B values than pure yellow. This can be a result of the non-ideal illumination system employed. Besides, no electronic color correction was employed. However, even with possible deviations from the color acquired the data of Fig. S1 show that the R and G components are basically constants as the dye concentration increases, and, more importantly, the B channel is linearly proportional with the 4-NP concentration. Therefore, there is no need for any type of image manipulation or color calibration. It is important to note that changes in the geometric arrangement slightly affect the slope of the intensity versus concentration profile. For instance, varying the distance between the camera and the reaction flask leads to different fitting parameters. However, the linear correlation between the dye concentration and the Blue channel remained (Fig. S2).

Calibration curves with flasks of different sizes were also performed. In this case, a higher sensitivity of the Blue channel as a function of the dye concentration is observed as the flask diameter increases (Fig. S3). The linear correlation is maintained, although the color saturation occurs at a lower concentration for larger diameters of the flasks containing the solution. Reproducibility was evaluated by preparing calibration curves in triplicate using fixed geometric arrangements. As seen in Fig. S4, the fitting parameters are very close assuring the reproducibility of the measurements.

The kinetic experiment, i.e., hydrolysis of 4-NPA with liberation of 4-NP was performed simultaneously in three vials using the setup described in the experimental section. The concentration of 4-NPA was 33 µmol L^−1^ and the pH was kept at 9.9 with a 0.01 mol L^−1^ carbonate/hydrogen carbonate buffer ensuring pseudo-first order conditions. The reaction solution was transferred to three reaction vials and the variation of intensity in the blue channel as a function of time was recorded as given in Fig. 2. For comparison, UV–Vis scans were recorded in parallel using an identical fresh solution.

As the reaction progresses the solutions become yellow due to the release of 4-NP. The variation in the blue channel as well as variations in the absorbance at 400 nm were fitted according to a first order reaction with coefficients of determination greater than 0.998. As can be seen, the method confirmed to be as accurate as UV–Vis spectrophotometry in view of the similarity between the mean first order rate constants obtained by the digital imaging (0.018543 min^−1^) with that of spectrophotometry (0.01848 min^−1^). Therefore, a difference of less than 1% in absolute value. The results obtained for the homogenous system encouraged us to study more challenging heterogeneous systems as discussed below.

### Heterogeneous kinetics monitoring

Three different heterogeneous kinetics were studied: case I is the reduction of 4-NP in the presence of dispersed Pd/C particles; case II is the photodegradation of methyl orange (MO) dye with H_2_O_2_ under UV light in the presence of TiO_2_ particles; case III is the adsorption of rhodamine dye on montmorillonite.

**Figure 3:**
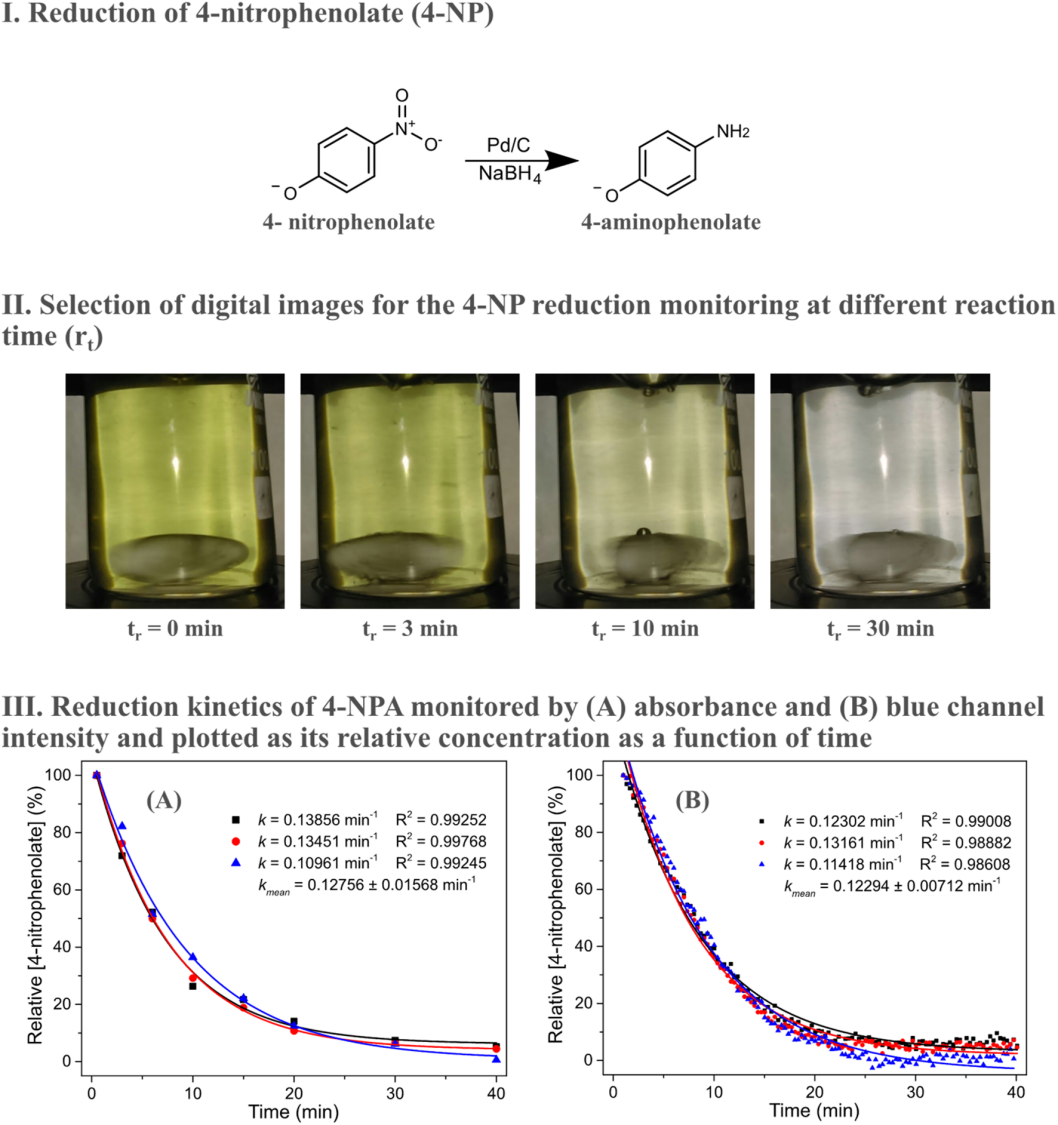
Reduction of 4-NP in the presence of Pd/C and NaBH_4_ (I). Selection of digital images captured in the kinetic monitoring of 4-NPA reduction (II). Relative variations of 4-NP concentration as a function of time as measured by UV–Vis at 400 nm (III-A) and by the blue channel intensity (III-B). Reactions performed in triplicate.

The reduction of 4-NP in the presence of excess NaBH_4_ and Pd/C particles is shown in Fig. 3. This model reaction is frequently used to probe the performance of novel hydrogenation catalysts. Kinetic monitoring of this reaction was performed in a 100 mL beaker with an initial substrate concentration of 11 µmol L^−1^. The choice of this considerably large volume when compared to the homogeneous hydrolysis of 4-NPA is due to the need to remove aliquots for spectrophotometric analysis. As the reaction advances the solution changes from yellow to colorless. Therefore the intensity of the blue channel as a function of time, shown in Fig. 3, is the opposite to that observed for 4-NPA hydrolysis. The data obtained with the smartphone were fitted according to a first order kinetics and the coefficients of determination were greater than 0.98. The monitoring methodology demonstrated to be straightforward and reproducible, presenting a standard deviation between measurements of only 5.79% for the rate constant, similarly to the homogeneous system (4.18%) indicating that the simple methodology reported herein can be used to monitor heterogeneous processes.

For the sake of comparison, 4-NP reduction kinetics was also followed by UV–Vis analysis by withdrawing aliquots of the solution monitored with the smartphone at different intervals. Samples were quickly centrifuged for 15 s at 6000 rpm and the supernatant was read at 400 nm. The main disadvantage in performing this classical approach lies in the need of sample processing, which increases experimental time and decreases analytical frequency. Besides, it is possible that during the time needed for sample preparation the reaction continued to proceed, which can ultimately result in unreliable measurements. In addition, a dedicated analyst is needed throughout the process.

As a case study II, the degradation of MO was monitored. Azo dyes are commonly found in wastewater from sectors including textiles, leather, pulp and paper, among others, causing enormous environmental impacts. Therefore, several degradation strategies have been studied, such as electrochemical oxidation^26^, degradation by enzymes^27^ and microorganisms^28^. In addition, advanced oxidative degradation processes are employed, especially in the treatment of stable chemicals^29^.

**Figure 4:**
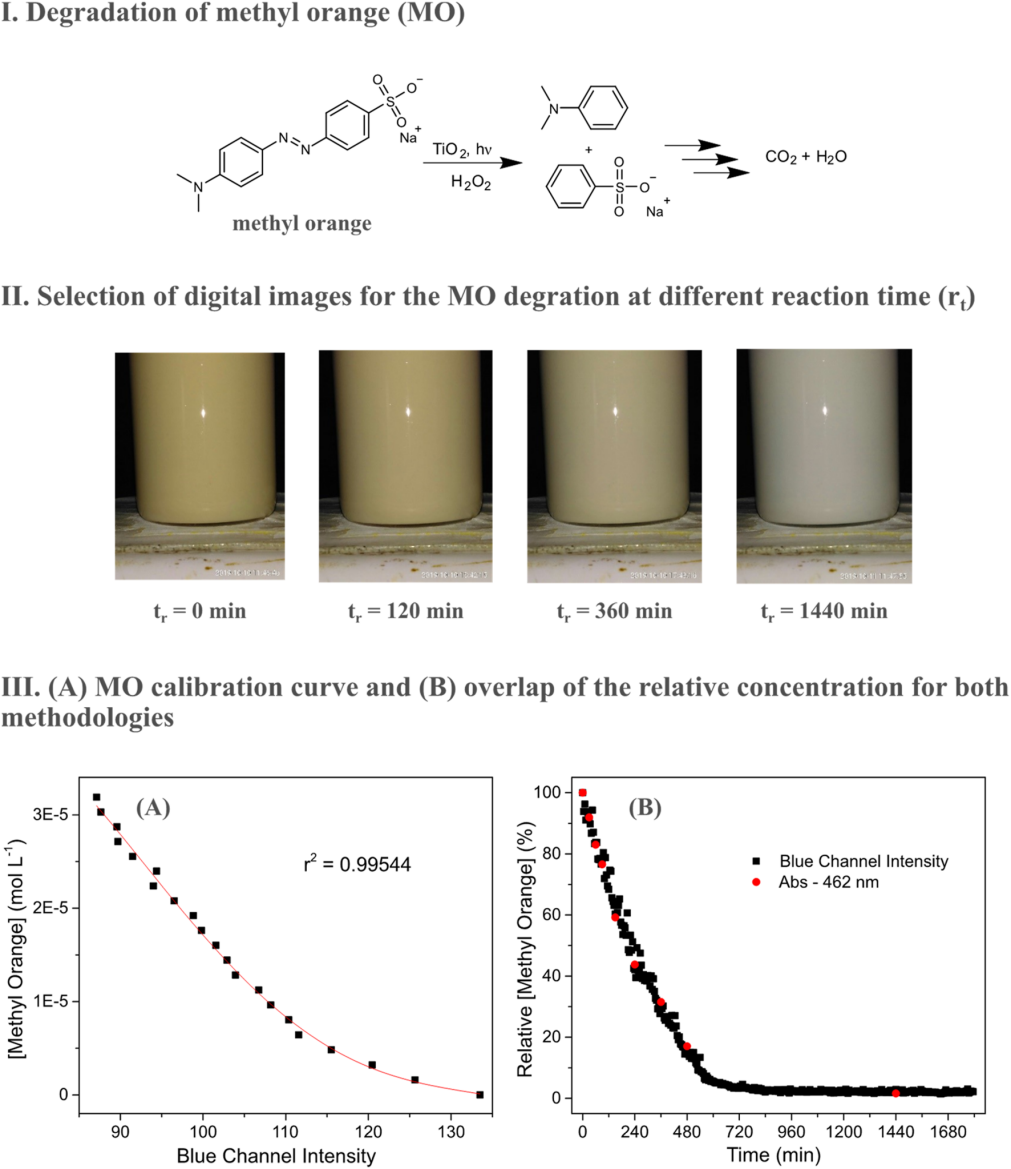
Degradation of MO with H_2_O_2_, TiO_2_ and UV light (I). Selection of digital images captured in the kinetic monitoring of MO degradation (II). MO calibration curve as a function of the intensity of the Blue channel (III-**A**). Overlapping of the relative MO concentration determined by both methodologies (III-**B**).

Herein, the degradation of MO was performed by hydrogen peroxide combined with TiO_2_ particles under UV light, as reported elsewhere^30^. Since the products do not absorb visible light, the solution changes from faint yellow to milk white due to the presence of small TiO_2_ suspended particles (Fig. 4).

Unlike previous systems containing 4-NP, none of the RGB channels correlates linearly with the concentration of MO in the solution. Consequently, it is necessary to associate one of the RGB channels to the dye concentration in the presence of TiO_2_ to interpret the kinetic data. In addition, to avoid possible interference in the data due to the variations caused by the visible light coming from the UV lamp, the calibration curve was prepared with the UV lamp on the same reaction flask used in the kinetic experiment. After the photos needed to prepare the calibration curve were taken, the reaction was initiated by the addition of the oxidizing reagent.

From the calibration curve images, a non-linear variation of the Blue channel is observed as a function of concentration. The curve adjustment was performed with an equation that best suited the data. In this specific case, a good correlation was observed with the fourth order polynomial equation (R^2^ = 0.99544). From this equation, the dye concentration over the course of the reaction can be calculated from the blue intensity of the photos. Analogous to case study I, reaction monitoring was also performed in parallel by UV–Vis, where an excellent correlation between the measurements of both methods was perceived (Fig. 4).

**Figure 5:**
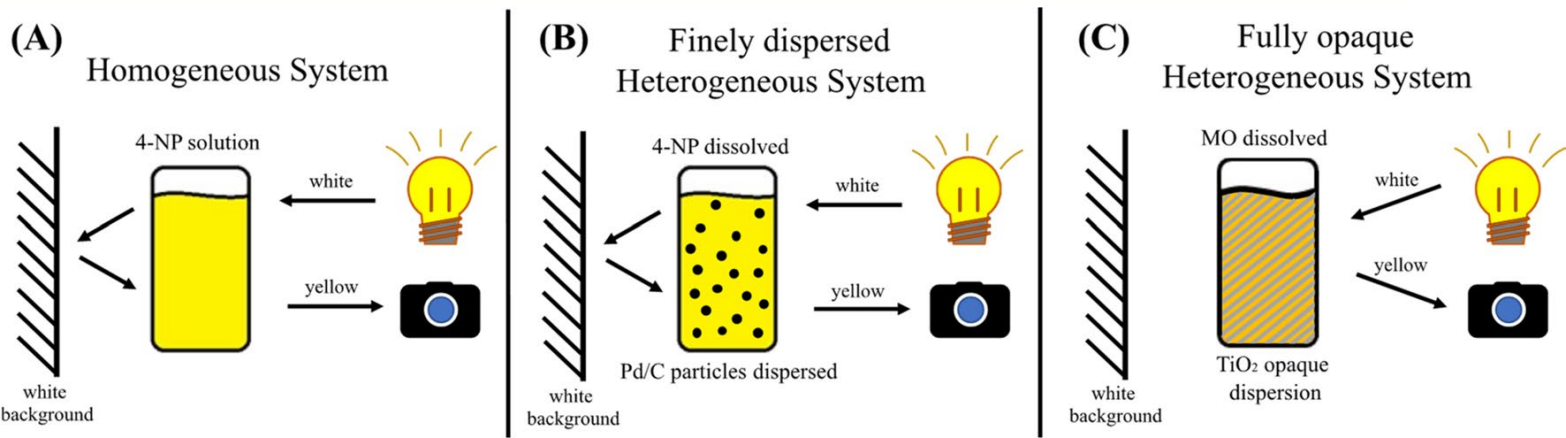
Representation of the three types of systems where the RGB channels could be correlated with the dye concentration.

Thus, we demonstrate that reaction kinetics of three types of systems, where the color observed is a result of transmitted and/or scattered light, can be conveniently monitored using a smartphone. In the homogeneous 4-NPA degradation the color observed corresponds to the transmitted light from the incident radiation that is partially absorbed by the dye in solution, reflects on the white background and reaches the observer/camera (Fig. 5A). This is also the mechanism involved in heterogeneous systems containing discrete dispersed particles such the 4-NP reduction with Pd/C (Fig. 5B). Conversely, for opaque media, such as for MO degradation in the presence of TiO_2_, the observed color is a result of the radiation scattered and partially absorbed by the sample (Fig. 5C).

**Figure 6:**
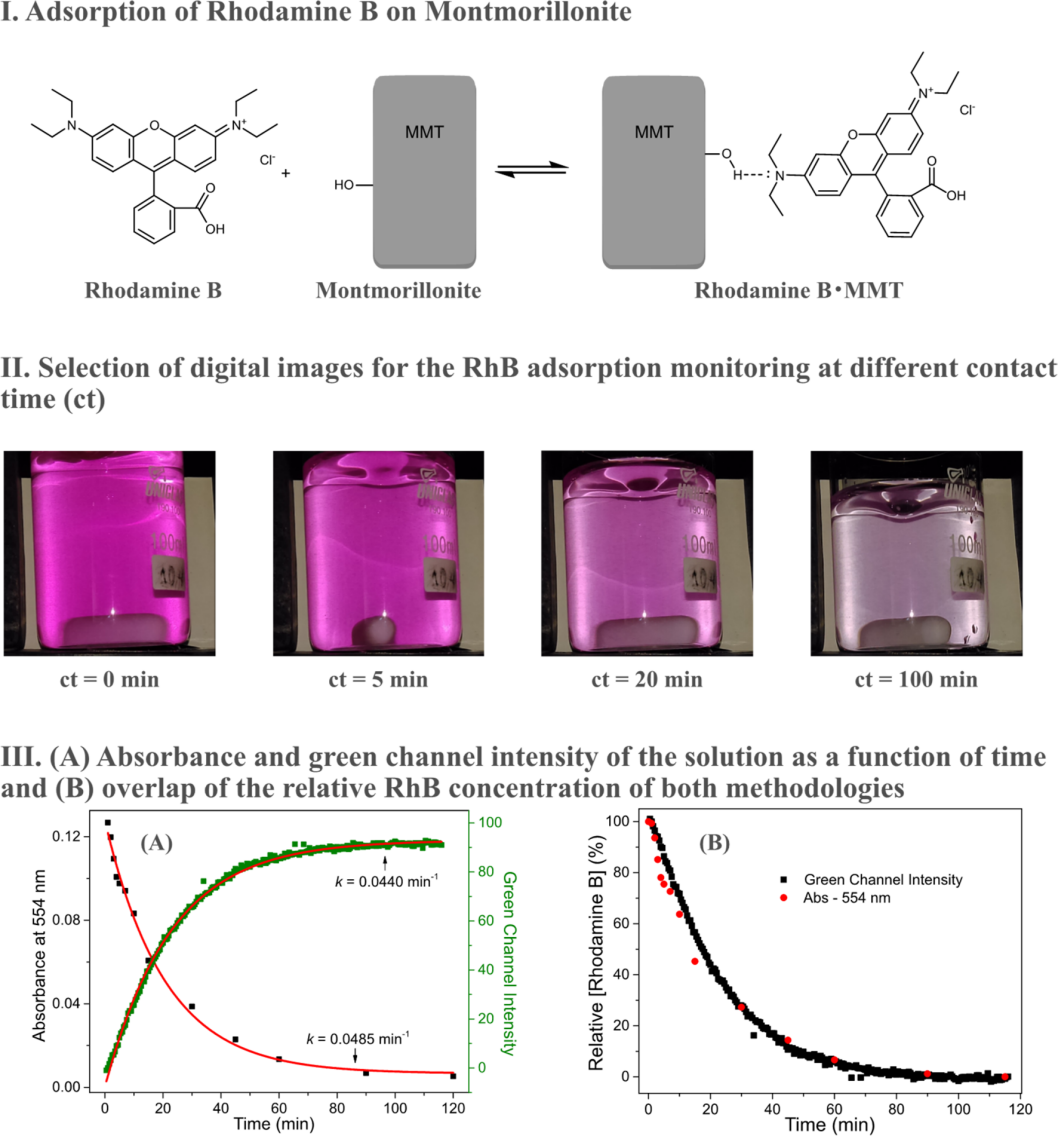
RhB adsorption on MMT surface (I). Selection of digital images captured in the kinetic monitoring of the adsorption process (II). Absorbance at 554 nm and Green intensity of digital images as a function of adsorption time (III-**A**). Overlapping of the relative concentration of RhB calculated by both methodologies (III-**B**).

Dyes that are stable to degradation processes can also be removed from effluents by adsorptive processes. Among adsorbents typically employed, montmorillonite is a popular choice due to its low cost and high adsorption capacity^31^. Therefore, as case study III, we monitored the adsorption kinetics of rhodamine B (RhB) on montmorillonite (MMT) simultaneously by digital images and by UV–Vis analysis (Fig. 6).

The concentration of RhB by digital images was evaluated by the Green channel because the intensity of this channel is linearly proportional to the concentration of the dye, as observed in Fig. S5. Both by the image and UV–Vis analysis the data are well defined by the pseudo-first order model, with adjusted R^2^ values greater than 0.99 for both cases. Very close rate constants were observed for the different methodologies, 0.0434 min^−1^ for the method proposed herein and 0.0485 min^−1^ for the traditional UV–Vis method, the subtle increase in the value of the second method possibly due to the short progression of the adsorptive process before and during the centrifugation step.

As the digital imaging methodology is equivalent in quantification compared to the UV–Vis methodology, the proposed method becomes more interesting than the classic approach due to the greater amount of data obtained during the reaction. Besides, it obviates the collection of samples and separation of solids for UV–Vis analysis. Importantly, the monitoring step is completely automated and therefore does not require a dedicated analyst or operator. In addition, the quantification of the dye is performed in real time, unlike the UV–Vis technique, where the time spent in the sampling process and in the separation of solids increases the risk that the measured concentration of the dye is discrepant with the actual concentration at the time of sampling.

## Conclusions

We have developed a generalizable and frugal computer-vision based system for kinetics monitoring. The initiative to use the intensity values of the RGB channels of digital images for kinetic monitoring of reactions in homogeneous and heterogeneous systems showed to be efficient. It facilitates the automation of common kinetic monitoring procedures where the inconveniences of the classical spectrophotometric methodology make the proposed approach via digital images more attractive.

The RGB analysis performed herein is equivalent to UV–Vis in terms of quantification power, however the former is more advantage due to the higher sampling frequency, allowing a better understanding of the reaction kinetic model. Besides, it does not require sampling and separation of particulates, allowing the process to be automated. Finally, the smartphone permits access to real-time analysis of the reaction progress. However, perhaps the inconvenient need to carry out a dye calibration curve prior to a reaction is possibly the greatest weakness of the methodology.

Our ability to easily measure and respond to dynamic situational changes are key to enabling and deploying flexible automation workflows. In addition, simplicity and low cost make the presented solution very accessible for educational purposes, (e.g., as in an experiment in undergraduate courses) and making science more equitable. On the other hand, the methodology has potential as an analytical tool in academic research as well as in industry.

## Experimental section

### Materials

4-NPA was synthesized as previously reported^32^. Anhydrous sodium carbonate P.A. (Vetec), Hydrogen Peroxide 35% (CRQ), MO (Sigma-Aldrich), Montmorillonite KSF (Sigma-Aldrich), 4-nitrophenol (Fluka Analytical), Palladium on carbon 9.7% (Sigma-Aldrich), Potassium hydroxide (Neon), Rhodamine B (Sigma-Aldrich), Sodium acetate P.A. trihydrate (Vetec), Sodium borohydride P.S. (Vetec) and Titanium (IV) oxide anatase (Sigma-Aldrich) were purchased with a high grade of purity.

### UV–Vis spectroscopy

UV–Vis analysis were carried out using a Varian Cary 50 UV–Vis spectrophotometer with quartz cuvettes.

### Data gathering and experimental workflow

**Figure 7:**
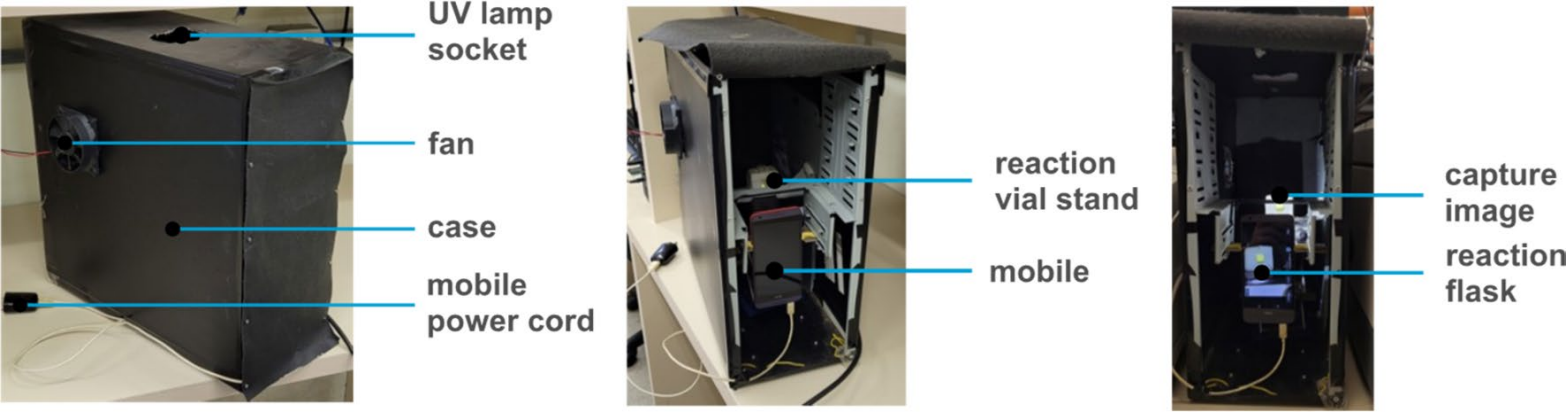
The exterior (left) and interior (middle and right) of the computer case used for capturing images.

The smartphone monitoring system was built inside a computer case to isolate the sample from exterior light (Fig. 7). A computer fan was attached to allow thermal equilibration with the room temperature (22.0 ± 1.0 °C). Inside the case a magnetic stirrer was placed on a lifting stand on which the reaction flask was positioned in front of the camera phone. For kinetic monitoring of MO photodegradation, as described later, a black light lamp was installed inside the system.

All results reported in this paper were obtained using an HTC Desire 626 s smartphone with 8-megapixel camera resolution. This phone detects radiation from 380 up to 1000 nm as determined using a manually operated monochromator. Similar results were obtained using different smartphones, the only requirement being a built-in flash as the lighting of the reaction system was accomplished by using the flash of the phone itself. Photo acquisition procedure is better detailed in Support Information. For the sake of comparison, spectrophotometric analyses were also performed. For the hydrolysis reaction of 4-nitrophenyl acetate (4-NPA) the reaction was performed directly in the cuvette, while for the reaction involving particulates aliquots were taken from the solutions while monitored by the smartphone, followed by centrifugation, and reading.

The workflow can be broken down into three stages: prepare, monitor, and process (Fig. 1). In the preparation stage, the researcher sets the experiment parameters and the region of interest to be monitored throughout the reaction. For image capture and data processing, we developed a program in Python to conduct the entire process (more details in Support Information), so the initial system setup is minimal. The researcher needs to provide the input values for initial parameters such as total monitoring time, time interval between photos and area of the image set considered for the analysis. Once started, the system will follow the programmed workflow, automatically updating values and iterating throughout the experiment until the process is complete or an interruption due to error or a stop request occurs. The automated reaction monitoring workflow described here was run via a master Python script with each module being controlled using custom code packages. As soon as photos are taken, a report that correlates the average RGB intensities from a selected area of the photos as a function of time is generated. For the mathematical treatment of the generated data the Origin software was used.

### Method reproducibility

Calibration curves were made from images generated with progressive addition of 10 µL of 1.58 mmol L^−1^ of 4-nitrophenol standard to a 3 mL solution of 0.01 mol L^−1^ KOH in a 1.6 mL diameter 5 mL flask. The procedure was performed in triplicate, with the vial kept at 15 cm from the smartphone.

The profile of the calibration curves was analyzed as a function of the variation of vial to camera distance and vial diameters parameters. Calibration curves were performed by varying the distance of 10, 15 and 20 cm from the 5 mL flask in relation to the camera. Also, calibration curves were made in a 1.6 cm diameter 5 mL-flask, in a 3.7 cm diameter 50 mL-beaker and in a 4.9 cm diameter 100 mL-beaker, where it was kept fixed at a 15 cm distance.

### Kinetic monitoring of 4-NPA hydrolysis

Hydrolysis kinetics of 4-NPA was performed in a 1.6 cm diameter vial by adding 20 µL of a 5 mmol L^−1^ solution of the ester in 3 mL of a 0.01 mol L^−1^ aqueous solution of sodium carbonate at pH 9.95. The reaction was monitored at room temperature with an image capture frequency of 2 photos/minute. Another reaction was carried out in parallel under the same conditions using a commercial UV–Vis spectrometer.

#### Kinetic monitoring of 4-NP reduction—case study I

A dispersion containing 9 mg of Pd/C 9.7% in 100 mL of 0.1 mol L^-1^ KOH and 0.011 mmol L^−1^ 4-NP was magnetically stirred for a minute in a 4.9 cm diameter beaker positioned 15 cm from the camera. An aliquot of an aqueous NaBH_4_ solution was added, giving an initial concentration 3 mmol L^−1^, and photos were taken at a frequency of 3 photos/minute. The reaction was performed in triplicate at room temperature. During the reaction 1 mL aliquots were taken, centrifuged at 6000 rpm for 15 s and then read on the UV–Vis spectrophotometer.

#### Kinetic monitoring of MO degradation—case study II

In a 4.9 cm diameter beaker kept 15 cm from the camera, 0.3 g of TiO_2_ anatase was dispersed in 100 mL of 0.01 M phosphate buffer at pH 7.2. The magnetic stirring and UV lighting from a Taschibra TKT 26-2 SHY 26 W lamp were activated. 50 µL aliquots of a 3.22 mmol L^−1^ MO solution were added up to a total of 1 mL, and images were captured after each addition. These initial photos were captured for the construction of the calibration curve. Finally, 3 mL of a 35% H_2_O_2_ aqueous solution were added, and the kinetics of MO degradation was monitored at a frequency of 1 photo/minute. Throughout the reaction, 1 mL aliquots were removed, centrifuged at 14,500 rpm for 15 min and then read on the UV–Vis spectrophotometer.

#### Kinetic monitoring of RhB adsorption—case study III

10 mg of Montmorillonite (MMT) were dispersed under magnetic stirring in a 4.9 cm diameter beaker containing 100 mL of water. Images are captured with a frequency of 2 photos/minute after adding 700 µL of a Rhodamine B (RhB) solution to give an initial dye concentration of 1.52 µmol L^−1^. During adsorption, 1 mL aliquots were removed and centrifuged at 14,500 rpm for 15 s and then read on a UV–Vis spectrophotometer.

## Supplementary Information

Supplementary Information 1.

Supplementary Video 1.

## Data Availability

Calibration curves of 4-NP with different setup settings, calibration curve of RhB, application description, instructions and rate equations used to fit the data can be found in the Supplementary Information S1. A video demonstration of the application running is given in the Supplementary Video S2. Additional data related to this paper may be requested from the authors.
