# Supplementary material for: Automated monitoring the kinetics of homogeneous and heterogeneous chemical processes using a smartphone
Source: Sci Rep. 2022 Sep 21;12:15774. doi: 10.1038/s41598-022-20123-9 (PMC9492685; doi:10.1038/s41598-022-20123-9)
Supplement: Supplementary file 1 — Supplementary Information 1. [file 41598_2022_20123_MOESM1_ESM.docx]

Automated Monitoring the Kinetics of Homogeneous and Heterogeneous Chemical Processes Using a Smartphone

Mateus H. Keller^1^, Raphaell Moreira^2^, and Bruno S. Souza^1*^

^1^Department of Chemistry, Federal University of Santa Catarina, Florianopolis, Brazil

^2^Department of Chemistry, The University of British Columbia, Vancouver, British Columbia, Canada

^*^bruno.souza@ufsc.br

Supplementary Information

|  | page |
| --- | --- |
| Figure S1. Selection of digital images used in 4-nitrophenolate calibration curves and intensity profile of RGB channels as a function of 4-nitrophenolate concentration. | S2 |
| Figure S2: Effect of camera to vial distance in 4-nitrophenolate calibration curves. | S2 |
| Figure S3: Effect of flask size in 4-nitrophenolate calibration curves. | S3 |
| Figure S4: Reproducibility of 4-nitrophenolate calibration curves. | S3 |
| Figure S5: Intensity profile for RGB channels as a function of Rhodamine B concentration in a 100ml (⌀=4.9 cm) flask. | S4 |
| Application Description and instructions | S5-S9 |
| Rate Equations | S10 |


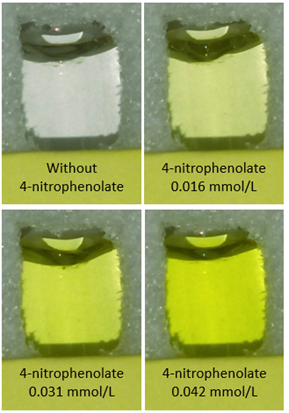


Figure S1: Selection of digital images used in 4-nitrophenolate calibration curves (left) and intensity profile of RGB channels as a function of 4-nitrophenolate concentration (right).

Figure S2: Effect of camera to vial distance in 4-nitrophenolate calibration curves. Samples were prepared in 5 ml (⌀=1.6 cm) vials.

Figure S3: Effect of flask size in 4-nitrophenolate calibration curves. Vial volumes and diameter: 5 mL (⌀=1.6 cm); 50 ml (⌀=3.7 cm); 100ml (⌀=4.9 cm).

Figure S4: Reproducibility of 4-nitrophenolate calibration curves. Red, blue and black lines are linear fittings to data collected in three separate experiments.

Figure S5: Intensity profile for RGB channels as a function of Rhodamine B concentration in a 100ml (⌀=4.9 cm) flask.

Application Description

The application developed by the authors can be found in our GitHub page (https://github.com/LACFI) in the .py extension and in our [Google Drive link](https://drive.google.com/drive/folders/1BeGP4EWRm9txOlG8LC-q-VOv8CmwXBYV?usp=sharing) in the .exe extension, along with the raw images and a demonstration video of the application running. The application has two main functionalities: (*i*) capturing images with a smartphone through a network connection and carrying out all subsequent data processing, or (*ii*) performing data processing on a set of images previously obtained and saved on the computer. Due to the advantages of automating the process through (*i*), this option is certainly the most attractive one. In any sense, users can use option (*ii*) if the imagens were captured using different applications.

For the connection between the Smartphotometer app on the computer and the smartphone, the free IP Webcam application (https://play.google.com/store/apps/details?id=com.pas.webcam) has been installed on the smartphone. Before starting the experiments, it is essential to configure this application on the user’s smartphone. Focus mode is set as “manual”, Flash mode is set as “Flashlight mode” and White Balance as “off”. It is of fundamental importance that the application used for capture does not adjust the focus, color or lighting of the photos during the capture process, as this can significantly alter RGB values ​​for an object of the same color. When the prerequisites are met, the user must start streaming and focus the image. On the mobile screen an IP address is shown (for example http://192.168.0.2:8080). This address is generated whenever a new streaming is started and must be copied for use in the Smartphotometer app. It is fundamental that the smartphone and the computer are connected to the same network. When opening the application, three main options are shown: Automated Process, Manual Process and Photo Set Process.

Automatized Process

With streaming started via IP Camera, the user can proceed to this function in the Smartphotometer app. The IP address must be typed in the first field as shown in the example and the Enter button must be clicked. Then the user has the option to click Get Frame. In this function, a capture is performed and shown to the user, so the image coordinates can be selected. In this image the user must click on two points and click on Enter on the keyboard. The first point must be upper and more to the left than the second. The procedure is exemplified in the image below. The coordinates obtained from the rectangle formed are presented in sequence. These values ​​are editable, so clicking Get Frame is completely optional. Then the user must click Browse to select the directory where a folder is created. Afterwards, the user must perform the following inputs: time interval between capturing photos and total capture time. To proceed to the next page the user clicks Next.


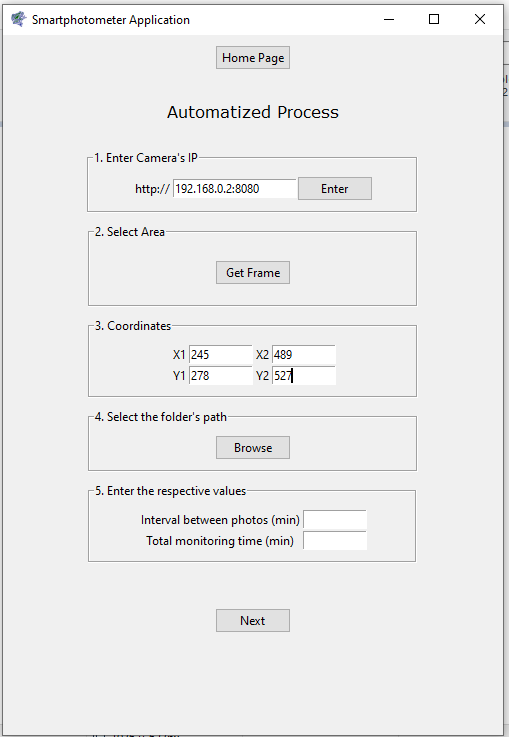

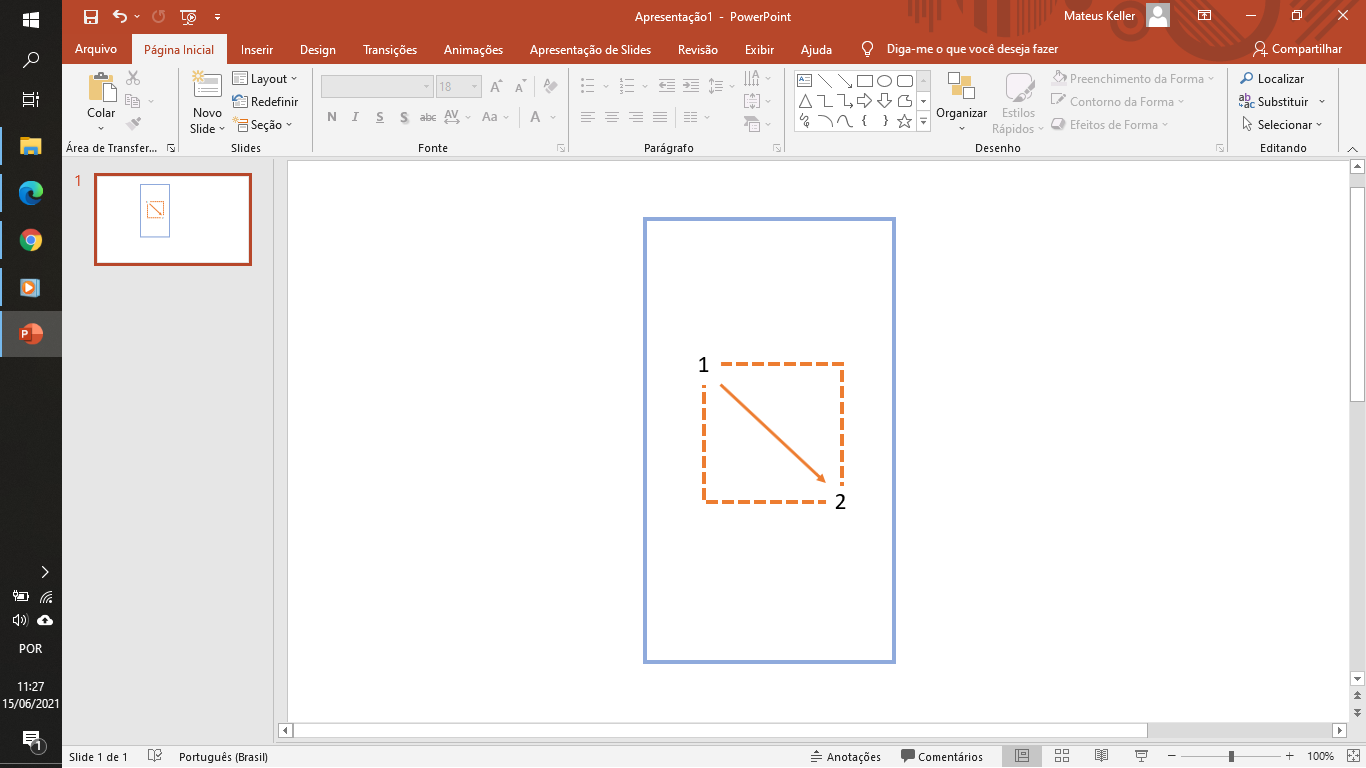


Figure S I: Main page of the Automated Process in the app (on the left). Example of the Get Frame step (on the right). An image is shown (blue rectangle) and the user clicks on two points, forming a rectangle represented in orange.

To start capturing photos, the user must click on Start Kinetics. During the predefined time, the program will capture photos, save them in the created folder, generate average RGB values for the images according to the stipulated area and present the RGB values to the user as a function of time. By pressing the buttons, the user can choose to view the RGB channel values altogether or individually as a function of time. When selecting a specific channel, the user can fit through the zero, first and second order rate equations by clicking the buttons (the equations are shown in the next section). A .txt file containing RGB values as a function of time is automatically generated in the folder created previously, and can be handled manually in other software, such as Excel or Origin.


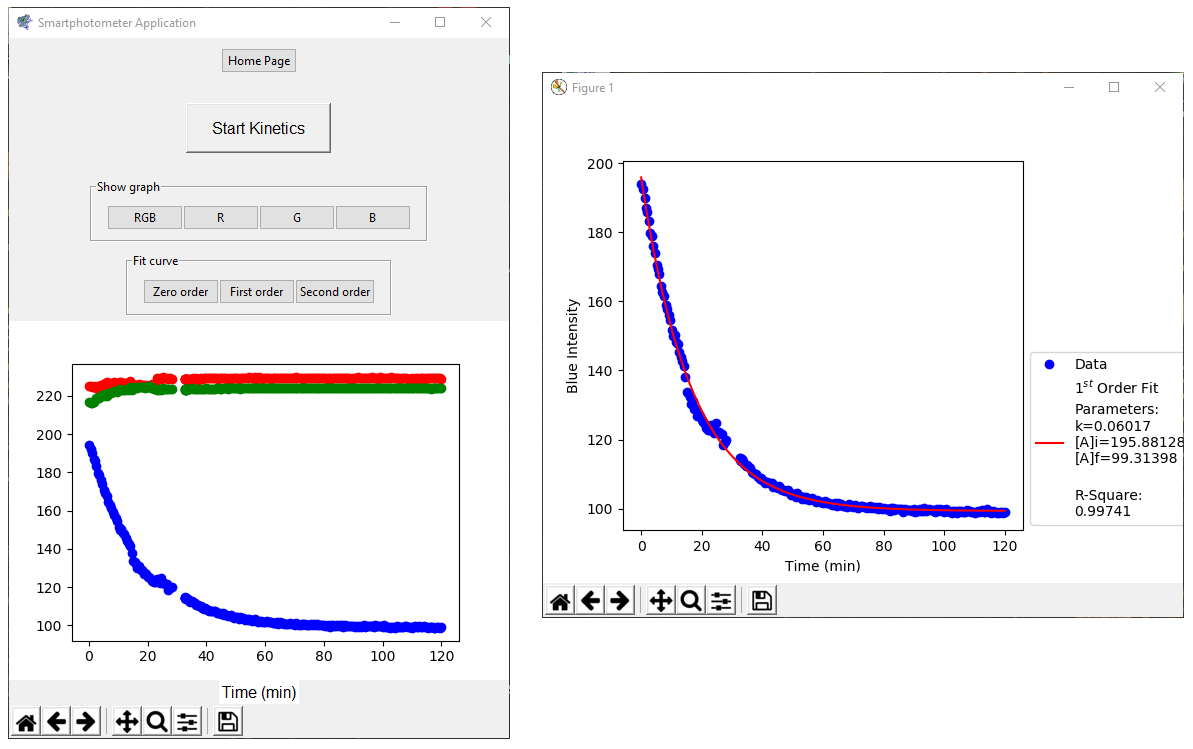


Figure S II: Second page of the Automated Process with data being processed in the app (on the left). First order fitting of the data by the app (on the right).

Manual Process

The operation of the Manual Process is basically the same as the Automated Process, with the difference that, instead of the program capturing images continuously at pre-established times, each image is captured by user command. This function is extremely useful for making calibration curves, for example. In this case, every time a dye solution is added to the main solution, the user captures a photo. Except for the “Δx” field, the first window is essentially the same as the previous process. The Δx value will be the increment on the axis, for example the volume of aliquots added to the solution. On the next page, the user can capture individual images in the Take Picture button. Likewise, the user can perform the linear adjustment through the program itself or work with the generated .txt file.


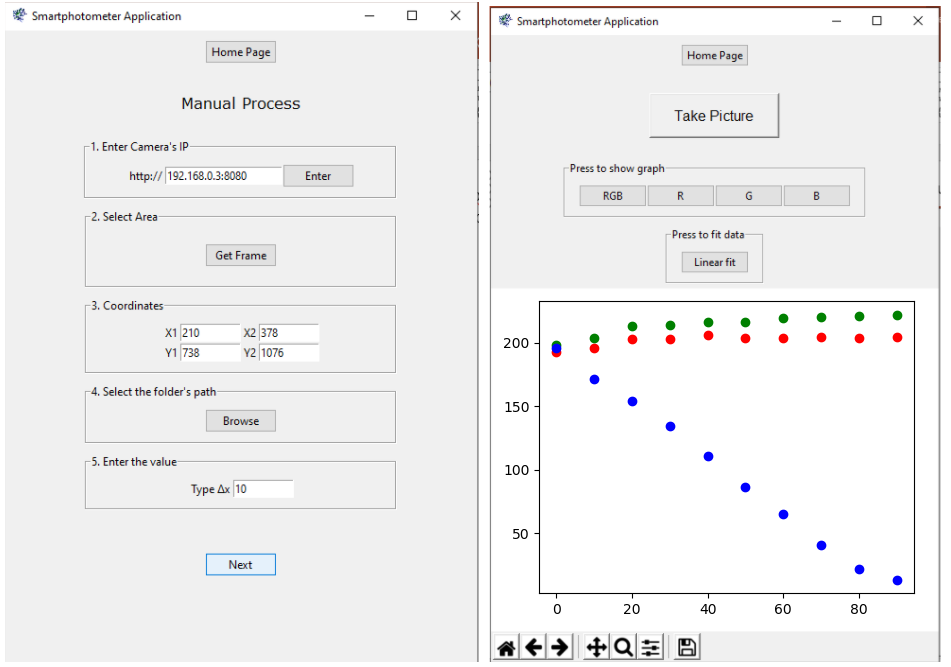


Figure S III: Main page (on the left) and Second page (on the right) of the Manual Process in the app.

Photo Set Process

When choosing the Photo Set function, the user must have previously taken photos saved in a folder in his/her computer. The set of photos could have been captured with this program or by any other method. When starting the Smartphotometer app in this function, the user must first select the folder with the photos, then enter the time interval between photos and click the Enter button. The next steps work in exactly the same way as in the Automated Process, where the user can fit the data with the zero, first and second order equations and can work with the generated .txt file. This function can also be used in making calibration curves, thus entering the Δx as input.


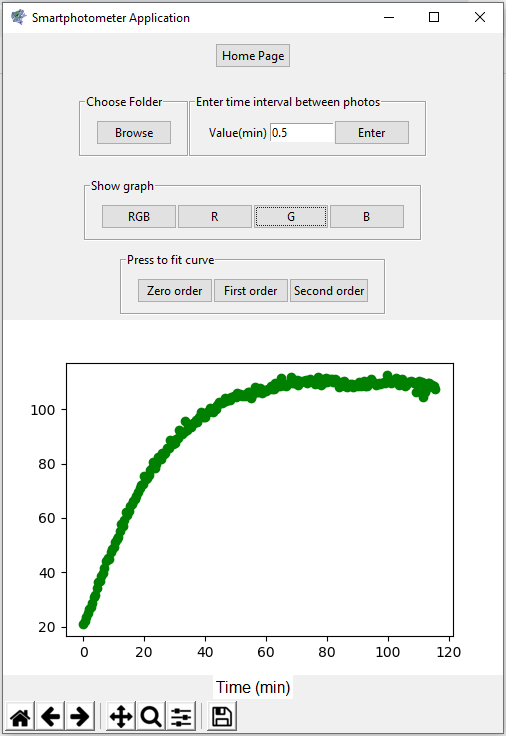


Figure S IV: Main page of the Folder Data Process in the app.

Rate Equations

Below are the corrected rate equations used by the software to fit the data. $[A]$ is taken as the intensity on one of the RGB channels. ${[A]}_{0}$ is the intensity calculated by the fitting for when the time is equal to zero. Similarly, ${[A]}_{f}$ is the channel intensity calculated for time tending to infinity.

Zero Order:

$$[A] = {[A]}_{0} - k\cdot t$$

First Order:

$$[A] = {[A]}_{f} + ({[A]}_{0}-{[A]}_{f}) \cdot e^{-k\cdot t}$$

Second Order:

When ${[A]}_{0}$ > ${[A]}_{f}$:

$$\left[ A \right]= \left[ A \right]_{f}+\frac{1}{\frac{1}{{[A]}_{0}-{[A]}_{f}}+k\cdot t}$$

When ${[A]}_{0}$ < ${[A]}_{f}$:

$$\left[ A \right]= \left[ A \right]_{f}-\frac{1}{\frac{1}{{[A]}_{f}-{[A]}_{0}}+k\cdot t}$$
